# Effectiveness and safety of transcatheter aortic valve replacement in elderly people with severe aortic stenosis with different types of heart failure

**DOI:** 10.1186/s12872-023-03048-7

**Published:** 2023-01-18

**Authors:** Mei Dong, Lizhen Wang, Gary Tse, Tao Dai, Lihong Wang, Zhicheng Xiao, Tong Liu, Faxin Ren

**Affiliations:** 1 Department of Cardiology, The Affiliated Yantai Yuhuangding Hospital of Qingdao University, Yantai, Shandong China; 2 Tianjin Key Laboratory of Ionic-Molecular Function of Cardiovascular Disease, Department of Cardiology, Tianjin Institute of Cardiology, Second Hospital of Tianjin Medical University, Tianjin, China; 3 Kent and Medway Medical School, Canterbury, CT2 7FS UK; 4 School of Nursing and Health Studies, Hong Kong Metropolitan University, Hong Kong, China; 5 Department of Ultrasound, The Affiliated Yantai Yuhuangding Hospital of Qingdao University, Yantai, Shandong China

**Keywords:** Transcatheter aortic valve replacement, Severe aortic stenosis, Heart failure with reduced ejection fraction, Heart failure with mildly reduced ejection fraction, Heart failure with preserved ejection fraction

## Abstract

**Background:**

Impaired left ventricular function is an independent predictor of adverse clinical outcomes in patients with aortic stenosis. The aim of this study is to evaluate the short-term changes of echocardiographic parameters, New York Heart Association (NYHA) class and B-type natriuretic peptide (BNP) level and adverse events amongst patients with heart failure (HF) after transcatheter aortic valve replacement (TAVR) procedure.

**Methods:**

This was a retrospective cohort study conducted at affiliated Yantai Yuhuangding Hospital of Qingdao University between September 2017 and September 2022. TAVR cases were stratified into three groups [heart failure with reduced ejection fraction (HFrEF), heart failure with mildly reduced ejection fraction (HFmrEF), heart failure with preserved ejection fraction (HFpEF)] by left ventricular ejection fraction (LVEF). Baseline characteristics, changes in echocardiographic parameters (1 week and 1 month), BNP (1 month), and NYHA class (6 months) post-TAVR were compared across the three groups. Meanwhile, we observed the adverse events of the patients after TAVR.

**Results:**

A total of 96 patients were included, of whom 15 (15.6%) had HFrEF, 15 (15.6%) had HFmrEF, and 66 (68.8%) had HFpEF. Compared to the HFpEF subgroup, patients in the HFrEF subgroup were younger (*p* < 0.05), and with a higher BNP (*p* < 0.05). The left ventricular end-diastolic dimension (LVEDD) in HFrEF group decreased significantly after TAVR. HFmrEF and HFrEF patients showed significant improvements in LVEF after TAVR. The pulmonary artery systolic pressure (PASP), aortic valve peak gradient (AVPG) and aortic valve peak gradient (V_max_) decreased significantly 1 month after TAVR in all three groups compared to the baseline (all *p* < 0.05). BNP significantly reduced in HFrEF group compared to HFpEF patients after TAVR (*p* < 0.05). The majority of patients experienced an improvement at least one NYHA class in all three groups 6 months post-TAVR. There is no significant increase in the risk of adverse events in the HFrEF group.

**Conclusions:**

Patients who underwent TAVR achieved significant improvements in BNP, NYHA class, LVEDD, LVEF, and PASP across the three HF classes, with a more rapid and pronounced improvement in the HFrEF and HFmrEF groups. Complication rates were low in the different HF groups. There is no significant increase in the risk of periprocedural complications in the HFrEF and HFmrEF groups.

## Introduction

The incidence of aortic stenosis (AS) increases with age and is a significant problem in an aging society [1–3]. Symptomatic severe aortic stenosis has dismal prognosis and early intervention is strongly recommended in all patients [1]. For patients undergoing surgical aortic valve replacement (SAVR) due to severe aortic stenosis, a reduced left ventricular ejection fraction (LVEF) is associated with increased mortality risk [4]. A reduced EF is one of the commonest reasons for patients being denied access to SAVR [5, 6]. Transcatheter aortic valve replacement (TAVR) is now increasingly offered to patients as an alternative to SAVR in patients with symptomatic severe AS and those with intermediate [7, 8] or higher [8–11] surgical risk and has been demonstrated to have comparable outcomes to SAVR.

Heart failure (HF) is a complex clinical syndrome with symptoms and signs that result from any structural or functional impairment of ventricular filling or ejection of blood [12]. AS and HF are two common causes of mortality in the elderly, they often coexist and affect one another. A Report of the Heart Failure Society of America, Heart Failure Association of the European Society of Cardiology, Japanese Heart Failure Society and Writing Committee of the Universal Definition of Heart Failure proposes a new and revised classification of HF according to LVEF: HF with reduced EF (HFrEF): HF with an LVEF of ≤ 40%; HF with mildly reduced EF (HFmrEF): HF with an LVEF of 41% to 49%; HF with preserved EF (HFpEF): HF with an LVEF of ≥ 50%; and HF with improved EF (HFimpEF): HF with a baseline LVEF of ≤ 40%, a ≥ 10-point increase from baseline LVEF, and a second measurement of LVEF of > 40% [13]. Prior research has demonstrated that impaired left ventricular function is associated with increased risks of adverse clinical outcomes in patients with severe aortic stenosis [14]. The use of TAVR in patients with HFrEF is controversial due to higher risks of complications [15]. Therefore, our study aims to evaluate the effects of TAVR on cardiac function among severe AS patients with different phenotypes of HF, accessing by echocardiographic parameters, B-type natriuretic peptide (BNP) level and New York Heart Association (NYHA) class. The incidence of adverse events was also evaluated across three groups to further elucidate the safety outcome of TAVR.

## Methods

### Study population

The study population included patients who underwent TAVR at the affiliated Yantai Yuhuangding Hospital of Qingdao University from September 2017 to September 2022. Our study complies with the Declaration of Helsinki. This study received ethics approval from Affiliated Yantai Yuhuangding Hospital of Qingdao University Ethics Committee. The ethics approval number is 2022–75. All patients provided written informed consent to participate. Patient inclusion criteria comprised: (1) underwent TAVR for symptomatic severe AS [mean gradient ≥ 40 mmHg (1 mmHg = 0.133 kPa), peak velocity ≥ 4.0 m/s, valve area ≤ 1 cm^2^ (or ≤ 0.6 cm^2^/m^2^) at the Affiliated Hospital of Qingdao university; (2) complicated with HF symptoms and/or signs, such as breathlessness, orthopnoea, paroxysmal nocturnal dyspnoea, reduced exercise tolerance, fatigue, inability to exercise and fluid retention [13]; (3) with a BNP level ≥ 35 ng/L or N-terminal pro-B-type natriuretic peptide (NT-proBNP) ≥ 125 ng/L. The exclusion criteria included (1) mild or moderate AS; (2) could not be measured by echocardiography; (3) severe rheumatic AS; (4) without HF. Patients were divided into 3 groups according to the baseline EF: reduced (≤ 40%; n = 15 ), mildly reduced (≥ 41% and ≤ 49%; n = 15), and preserved (≥ 50%; n = 66) EF.

### TAVR procedure and medication

All patients underwent preoperative investigations such as computed tomography (CT) scan, electrocardiogram and transthoracic echocardiography. All patients underwent transfemoral access and general anesthesia for the TAVR procedure. The choice of prosthesis and the performance of pre- or post-dilation was left to the interventionalist’s discretion [16]. Unfractionated heparin was used for periprocedural anticoagulation. Suture-mediated closure devices were used for access-site closure [16].

### Data collection

Baseline characteristics for each participants were collected, including demographics (age, sex, body mass index), symptoms (angina, dyspnea, syncope), NYHA class, smoking, heart rate, medical comorbidities such as hyperlipidemia, diabetes, dyslipidemia, chronic kidney disease (CKD), coronary heart disease (CHD), prior percutaneous coronary intervention (PCI) or coronary artery bypass grafting (CABG) history, history of myocardial infarction (MI), stroke/transient ischemic attack (TIA) and atrial fibrillation (AF). The improvement of NYHA class is evaluated according to the guiding principles of chronic heart failure (CHF) clinical research. Invalid NYHA change was defined as no change in NYHA class or deterioration in NYHA class. Effective NYHA change was defined as improvement of 1 NYHA class(es). Remarkable effect NYHA change was defined as improvement of 2/3NYHA class(es). Laboratory examination included BNP, high-sensitivity Troponin I(hsTnI), uric acid, homocysteine, blood lipid, hepatic function, creatinine, urea and blood counts. Transthoracic echocardiography was performed before and after TAVR procedure. All echocardiograms were obtained with the patient in a stable hemodynamic condition. Echocardiographic parameters included left atrium anteroposterior diameter (LAAD), left ventricular end-diastolic dimension (LVEDD), right ventricular anteroposterior diameter (RVAD), left ventricular end-diastolic volume (LVEDV), aortic valve peak gradient (AVPG), aortic valve mean gradient (AVMG), peak aortic velocity (V_max_), pulmonary artery systolic pressure (PASP), LVEF, moderate-severe aortic regurgitation (AR) and moderate-severe tricuspid regurgitation (TR). Moderate-severe valve regurgitation includes moderate valve regurgitation and severe valve regurgitation. According to the valve regurgitation guidelines [17], the degree of valve regurgitation was comprehensively assessed using echocardiographic qualitative, semi-quantitative, and quantitative indicators. LVEF was assessed by the Simpson’s method. TAVR cases compared for the incidence of adverse events over a 6-month follow-up. Adverse events include vascular complications, heart block, AF, poor wound healing, secondary thoracotomy for hemostasis, coronary obstruction, perivalvular leakage, permanent pacemaker, stroke and all-cause mortality. Patients underwent baseline laboratory examination and echocardiography before TAVR and regular examinations 1 week, 1 month, 6 months, one year and every year thereafter after TAVR according to a standard follow-up protocol. Adverse events data were obtained from medical records or by inquiring the patients' families or referring physicians through telephone. The duration of follow-up in this study is 6 months.

### Statistical analysis

Data analysis was performed using SPSS (version 25.0). Categorical variables were expressed as absolute numbers and percentages. The Chi-squared test or Fisher’s exact test was used compare categorical variables between groups. Data normality was assessed using the Shapiro–Wilk test. Normally distributed continuous variables were presented as mean and SD. Paired t-tests and ANOVA were used to compare normally distributed continuous variables, followed by a post hoc analysis performed using the Scheffe test. Non-normally distributed continuous variables are presented as median with 25%-75% interquartile range. We use the nonparametric Kruskal Wallis test to compare non-normally continuous variables. A 2-tailed *p* < 0.05 was considered statistically significant.

## Results

A total of 96 patients underwent TAVR for severe AS were included. Amongst these, 66 patients were classified as HFpEF, 15 as HFmrEF and 15 as HFrEF. Baseline characteristics are demonstrated in Table 1. The mean age of the groups decreased with increasing severity of EF reduction (*p* < 0.05). Compared to patients with HFpEF, those with HFrEF were significantly younger (*p* < 0.05). Besides, NYHA class at baseline was significantly higher in patients with HFrEF. In patients with HFrEF, 46.7% were male, with a mean age of 69.13 ± 6.79. The mean body mass index (BMI) was 22.1 kg/m^2^. In terms of cardiovascular risk factors, 33.3% were identified as smokers, and the prevelance of hypertension, diabetes, dyslipidemia and CKD were found to be 46.7%, 13.3%, 33.3% and 6.7% respectively, in HFrEF patients. Baseline cardiovascular disease including CHD (73.3%), prior MI (20.0%), AF (6.7%) and stroke (6.7%) were captured. Baseline cardiac Surgery included CABG (0.0%) and PCI (13.3%). There was no significant difference in heart rate between HFrEF and the other two groups. Compared to patients with HFpEF, hsTnI, BNP and uric acid at baseline was significantly higher in patients with HFrEF. Other laboratory parameters did not differ significantly across the three groups.

**Table 1:** Baseline characteristics of the study population [$\overline{X}$ ± S or M(P25, P75), case(%)]

Variable	HFrEF	HFmrEF	HFpEF	χ^2^, F or H	P
n	15	15	66		
Age (years)	69.13 ± 6.79^a^	71.07 ± 6.11	74.29 ± 6.16	4.982	0.009
BMI (kg/m^2^)	22.06 (20.76,27.14)	24.91 (21.05,28.34)	24.91 (22.72,27.55)	3.213	0.201
Women	7 (46.7)	4 (26.7)	34 (51.5)	3.031	0.220
Angina	6 (40.0)	3 (20.0)	21 (31.8)	1.403	0.499^∆^
dyspnea	13 (86.7)	13 (86.7)	41 (62.1)	5.562	0.054^∆^
syncope	0 (0.0)	1 (6.7)	8 (12.1)	1.637	0.568^∆^
NYHA class	^a^			17.259	0.001^∆^
II	0 (0.0)	0 (0.0)	5 (7.6)		
III	3 (20.0)	8 (53.3)	46 (69.7)		
IV	12 (80.0)	7 (46.7)	15 (22.7)		
smoking	5 (33.3)	5 (33.3)	15 (22.7)	1.449	0.526^∆^
Hypertension	7 (46.7)	11 (73.3)	41 (62.1)	2.291	0.318
Diabetes	2 (13.3)	6 (40.0)	16 (24.2)	2.754	0.278^∆^
Dyslipidemia	5 (33.3)	5 (33.3)	27 (40.9)	0.500	0.779
CHD	11 (73.3)	6 (40.0)	37 (56.1)	3.389	0.184
CKD	1 (6.7)	2 (13.3)	2 (3.0)	3.091	0.174^∆^
Prior MI	3 (20.0)	1 (6.7)	8 (12.1)	1.246	0.636^∆^
stroke	1 (6.7)	1 (6.7)	9 (13.6)	0.579	0.697^∆^
AF	1 (6.7)	4 (26.7)	11 (16.7)	2.041	0.338^∆^
CABG	0 (0.0)	0 (0.0)	2 (3.0)	0.576	1.000^∆^
PCI	2 (13.3)	1 (6.7)	12 (18.2)	0.987	0.755^∆^
heart rate (bpm)	87.00 (73.00, 95.00)	71.00 (62.00, 84.00)	71.50 (63.00, 82.00)	5.902	0.052
hsTnI (pg/ml)	63.50 (39.40,852.94)^a^	121.60 (43.00,166.40)^a^	23.40 (10.93,69.68)	17.615	0.000
BNP (pg/ml)	2880.85 (1275.48,4290.55)^a^	1487.91 (824.85,2370.30)^a^	432.17 (169.15,1071.94)	30.842	0.000
uric acid (umol/L)	512.00 (415.00, 639.00)^a^	448.00 (348.00, 559.00)	405.00 (282.00, 468.00)	10.326	0.006

BMI: Body mass index; NYHA: New York heart association; CHD: Coronary heart disease; CKD: Chronic kidney disease; MI: Myocardial infarction; AF: Atrial fibrillation; CABG: Coronary artery bypass surgery; PCI: Percutaneous coronary intervention; hsTnI: high-sensitivity Troponin I; BNP: B-type natriuretic peptide. ^∆^Fisher’s exact tests. ^a^Compared with HFpEF group, *p* < 0.05

The baseline echocardiographic findings are detailed in Table 2. LVEDD, LVEDV, LAAD and moderate-severe TR were significantly different across the 3 groups (*p* < 0.05). The median of preoperative LVEF of the three groups (HFrEF, HFmrEF, HFpEF) were 36.00, 46.00 and 62.00 respectively.

**Table 2:** Echocardiography at baseline [$\overline{X}$ ± S or M(P25, P75), case(%)]

Variable	HFrEF	HFmrEF	HFpEF	χ^2^, F or H	*p*
n	15	15	66		
LAAD(mm)	46.43 ± 4.64^a^	46.53 ± 5.33^a^	42.73 ± 6.22	4.192	0.018
LVEDD (mm)	60.29 ± 6.83^ab^	55.00 ± 6.00^a^	47.95 ± 6.66	24.721	0.000
RVAD(mm)	24.00 (21.00,26.00)	23.00 (22.00, 26.00)	24.00 (21.00,25.00)	0.246	0.884
LVEDV(ml)	203.00 (162.00,279.00)^a^	170.00 (132.00, 220.00)	141.00 (112.50,168.00)	12.036	0.002
AVPG (mmHg)	87.00 (68.00,111.00)	89.00 (57.00, 113.00)	94.00 (74.00,113.75)	2.230	0.328
AVMG(mmHg)	49.00 (38.00,60.00)	60.00 (28.00,71.00)	55.50 (45.00,69.50)	4.014	0.134
LVEF(%)	36.00 (34.00,37.00)	46.00 (42.00,47.00)	62.00 (57.00,66.00)		
Vmax (m/s)	4.51 ± 0.70	4.44 ± 1.03	4.87 ± 0.69	2.896	0.060
PASP(mmHg)	61.00 (42.00,75.00)	53.50 (37.25,66.00)	46.00 (30.25,62.75)	4.461	0.107
Moderate-severe AR(%)	6 (40.0)	8 (53.3)	22 (33.3)	2.133	0.344
Moderate-severe TR(%)	3 (20.0)	7 (46.7)^a^	5 (7.6)	12.369	0.001^∆^

LAAD: left atrium anteroposterior diameter; LVEDD: left ventricular end-diastolic dimension; RVAD: right ventricular anteroposterior diameter; LVEDV: left ventricular end-diastolic volume; AVPG: aortic valve peak gradien; AVMG: aortic valve mean gradient; LVEF: left ventricular ejection fraction; Vmax: peak aortic velocity; PASP: pulmonary artery systolic pressure; AR: aortic regurgitation; TR: tricuspid regurgitation. Moderate-severe valve regurgitation includes moderate valve regurgitation and severe valve regurgitation. ^a^Compared with HFpEF group, *p* < 0.05. ^b^Compared with HFmrEF group, *p* < 0.05. ^∆^ Fisher’s exact tests

The comparison of echocardiography parameters before and after operation is shown in Table 3. Compared with baseline, LVEF of the HFmrEF and HFrEF group increased significantly 1 week after TAVR (*p* < 0.05 for both). One month after TAVR, LVEDD in patients with HFrEF and HFpEF decreased significantly compared with baseline values (*p* < 0.05). And LVEF increased evidently in three groups 1 month post-TAVR (*p* < 0.05). In the HFmrEF group, 12 patients had increased LVEF 1 week post-TAVR, of whom 10 patients had a recovered LVEF level of ≥ 50%. The LVEF of eight patients in the HFrEF group increased to ≥ 41%, of whom 2 patients had recovered to ≥ 50%. One month after TAVR, three patients in HFmrEF group lost follow-up. In HFmrEF group, LVEF of 10 patients recovered to ≥ 50%. In HFrEF group, one patient was lost follow-up and one patient died during perioperative period. The LVEF of 11 patients in the HFrEF group increased to ≥ 41%, of whom eight patients recovered to ≥ 50%.

**Table 3:** Comparison of pre-and post-TAVR echocardiogram parameters for 1 week and 1 month [$\overline{X}$ ± S or M(P25, P75)]

Group	n	Before TAVR		1 week after TAVR		
		LVEDD(mm)	LVEF(%)	LVEDD(mm)	LVEF(%)	△LVEF(%)^d^
HFrEF	14	59.65 (55.00,62.25)	35.57 ± 2.98	57.00 (54.00,63.25)	43.07 ± 5.76^c^	7.50 ± 4.62^a^
HFmrEF	15	56.00 (51.00,59.00)	45.40 ± 2.80	54.00 (50.00,58.00)	52.93 ± 6.94^c^	7.53 ± 7.68^a^
HFpEF	63	48.00 (43.00,51.50)	61.37 ± 6.23	47.00 (43.60,51.00)	62.51 ± 5.15	1.14 ± 5.96

Group	n	Before TAVR		1 month after TAVR		
		LVEDD(mm)	LVEF(%)	LVEDD(mm)	LVEF(%)	△LVEF(%)^d^
HFrEF	13	60.11 ± 6.26	35.54 ± 3.10	54.39 ± 7.20^c^	50.08 ± 7.57^c^	14.54 ± 7.50 ^a^
HFmrEF	12	53.58 ± 5.87	45.42 ± 2.91	50.11 ± 6.71	55.00 ± 6.38^c^	9.58 ± 8.04^a^
HFpEF	53	48.09 ± 5.54	61.09 ± 6.28	45.89 ± 5.96^c^	65.42 ± 5.60^c^	4.32 ± 7.23

LVEDD: left ventricular end-diastolic dimension; LVEF: left ventricular ejection fraction. △LVEF is obtained by subtracting preoperative parameter from postoperative parameter. ^c^Compared with preoperative echocardiogram parameters, *p* < 0.01. ^a^Compared with HFpEF group, *p* < 0.05. ^d^Comparison of △LVEF among three groups, *p* < 0.01

The changes in echocardiographic parameters 1 week after TAVR across the three groups are shown in Tables 3 and 4. AVPG and Peak aortic velocity decreased in the three groups compared with baseline. Compared to patients with HFpEF, LVEF at 1 week was significantly improved in patients with HFrEF and HFmrEF. PASP in patients with HFrEF decreased significantly after TAVR.

**Table 4:** Changes of echocardiographic indexes 1 week and 1 month after TAVR [$\overline{X}$ ± S or M(P25, P75)]

Group(1 week)	n	△LAAD(mm)	△LVEDD(mm)	△RVAD(mm)	△LVEDV(ml)	△AVPG(mmHg)	△Vmax(m/s)	△PASP(mmHg)
HFrEF	14	1.28 ± 7.40	1.00 (− 0.25,2.25)	0.36 ± 2.13	19.00 (− 22.00,24.00)	63.00 (45.00,84.00)	2.60 (1.60,2.85)	23.00 (7.00,39.00)
HFmrEF	15	0.40 ± 4.12	2.00 (− 3.00,3.00)	0.07 ± 2.28	0.00 (− 18.25,27.50)	67.00 (38.25,97.75)	2.70 (1.80,3.20)	5.00 (− 3.00,29.50)
HFpEF	63	0.16 ± 4.97	0.00 (− 2.00,3.00)	0.00 ± 2.97	0.50 (− 29.25,37.50)	69.50 (60.75,95.50)	2.60 (2.20,3.20)	6.00 (1.00,23.00)
F or H		0.256	1.138	0.100	0.223	3.513	1.850	5.705
P		0.774	0.566	0.905	0.894	0.173	0.397	0.058

Group(1 month)	n	△LAAD(mm)	△LVEDD(mm)	△RVAD(mm)	△LVEDV(ml)	△AVPG(mmHg)	△Vmax(m/s)	△PASP(mmHg)
HFrEF	13	5.57 ± 7.15	5.72 ± 4.24	0.07 ± 2.06	6.67 ± 76.10	73.00(45.00,90.00)	2.39 ± 0.81	30.92 ± 16.64^ab^
HFmrEF	12	2.32 ± 7.20	3.48 ± 7.97	0.53 ± 2.82	− 5.58 ± 61.63	68.00(35.00,86.00)	2.13 ± 0.86^a^	13.80 ± 14.88
HFpEF	53	1.90 ± 4.25	2.20 ± 4.84	− 0.02 ± 3.57	7.90 ± 54.57	70.50(60.75,102.25)	2.78 ± 0.71	15.37 ± 17.01
F or H		1.524	3.290	0.137	0.254	3.130	4.384	4.381
P		0.245	0.058	0.873	0.776	0.209	0.016	0.017

LAAD: left atrium anteroposterior diameter; LVEDD: left ventricular end-diastolic dimension; RVAD: right ventricular anteroposterior diameter; LVEDV: left ventricular end-diastolic volume; AVPG: aortic valve peak gradien; AVMG: aortic valve mean gradient; Vmax: peak aortic velocity; PASP: pulmonary artery systolic pressure. △LAAD, △LVEDD, △RVAD, △LVEDV, △AVPG, △Vmax and △PASP are all obtained by subtracting postoperative parameters from preoperative parameters. ^a^Compared with HFpEF group, *p* < 0.05. ^b^Compared with HFmrEF group, *p* < 0.05

The changes in echocardiographic parameters 1 month after TAVR across the three groups are also shown in Tables 3 and 4. There were statistically significant differences in the improvement of peak aortic velocity, PASP and LVEF at 1 month across the three groups. Compared to HFpEF group, HFmrEF and HFrEF patients showed significant improvements in LVEF. Peak aortic velocity decreased significantly in HFpEF patients than in HFmrEF patients (*p* < 0.05). Compared with HFpEF and HFmrEF group, PASP in HFrEF group decreased more significantly after TAVR (*p* < 0.05). Compared to baseline, AVPG decreased significantly in the three groups 1 month post-TAVR (*p* < 0.05), without any significant inter-group differences.

**Fig. 1:**
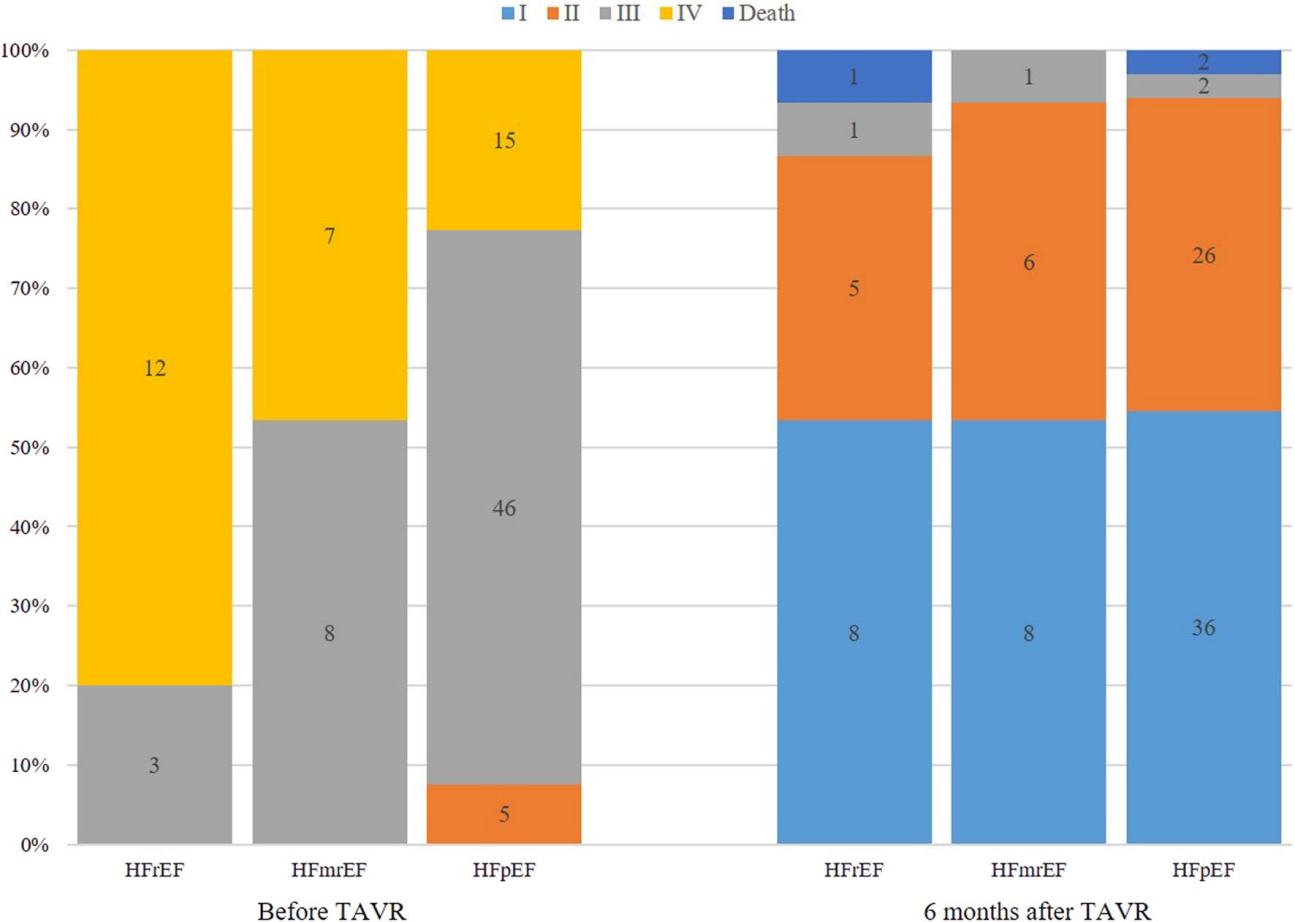
Changes of NYHA class before and after TAVR

The 1-month BNP changes after TAVR between the three groups are compared in Table 5. The BNP level of the HFmrEF and HFrEF groups was significantly decreased compared with group HFpEF (*p* < 0.05 for both). Subjective outcomes in terms of the NYHA class were collected during follow-up. Table 5 and Fig. 1 show the changes of NYHA class before and after surgery. Most patients were NYHA class III or IV at baseline and NYHA class I or II at follow-up.There was a similarly strong improvement of at least one NYHA class in all three groups, with 100.0, 93.4, and 93.8% for HFrEF, HFmrEF and HFpEF patients, respectively. However, there were no statistically between-group difference.

**Table 5:** Changes of BNP at 1 month and NYHA class at 6 months after TAVR [M(P25, P75), case(%)]

Variable	HFrEF	HFmrEF	HFpEF	H, χ^2^	P
n	14	15	64		
△BNP(pg/ml)	1824.40 (1038.20,3028.21)^a^	953.09 (483.55, 1628.83)^a^	143.39 (6.12,774.84)	25.637	0.000
NYHA class				5.637	0.173^∆^
Remarkable effect	12 (85.7)	13 (86.7)	40 (62.5)		
Effective	2 (14.3)	1 (6.7)	20 (31.3)		
invalid	0 (0.0)	1 (6.7)	4 (6.3)		
effective rate(%)	100	93.4	93.8		

NYHA: New York heart association; BNP: B-type natriuretic peptide. △BNP is obtained by subtracting postoperative parameter from preoperative parameter. ^a^Compared with HFpEF group, *p* < 0.05. ^∆^Fisher’s exact tests. 3 patients died during the perioperative period

The intraoperative and postoperative clinical details are shown in Table 6. Longer intensive care time were observed for the HFrEF group (*p* < 0.05). And HFrEF and HFmrEF groups have longer length-of-stay of hospitalizations.

**Table 6:** Intraoperative and postoperative clinical data [M(P25, P75)]

Variable	HFrEF	HFmrEF	HFpEF	H	P
n	15	15	66		
Operation time (min)	170.00 (130.00,190.00)	135.00 (120.00, 180.00)	137.50 (120.00, 170.00)	3.431	0.180
Intraoperative blood loss (ml)	50.00 (50.00,100.00)	50.00 (20.00,100.00)	50.00 (20.00,100.00)	1.233	0.540
Intensive care time (h)	69.86 (44.81, 93.32)^a^	44.72 (22.22, 141.85)	44.60 (23.92, 50.19)	8.378	0.015
Hospital stay time (d)	25.00 (16.00, 29.00)	26.00 (16.00, 37.00)^a^	17.00 (14.00, 23.25)	9.490	0.009

^a^Compared with HFpEF group, *p* < 0.05

Post-TAVR outcomes are shown in Table 7. Left bundle branch block (LBBB) was the most common type of conduction disturbances induced by TAVR in this study, with a prevalence of 16.7% in the overall study population. In patients with HFrEF, HFmrEF and HFpEF, the prevalence of LBBB was 6.7%, 6.7% and 21.2%, respectively. Other arrhythmias induced by TAVR in AS patients with HF included complete heart block (8.3%), and atrial fibrillation (4.2%). Among the overall study population, 8 patients (8.3%) had implanted a permanent pacemaker due to complete heart block. The incidence of adverse events was low in the three groups. In terms of all-cause mortality post-TAVR, only one death (6.7%) occurred in the HFrEF subgroup, due to ventricular fibrillation. Two other deaths (3.0%) were observed in the HFpEF subgroup because of coronary obstruction during TAVR. Of the total study population, other adverse events included vascular complications (3.1%), poor wound healing (1.0%), secondary thoracotomy for hemostasis (1.0%), moderate valve regurgitation (6.3%), and stroke (5.2%).

**Table 7:** Postoperative adverse events [case (%)]

Variable	HFrEF	HFmrEF	HFpEF	Total study population
n	15	15	66	96
Vascular complications	0 (0.0)	0 (0.0)	3 (4.5)	3 (3.1)
New onset CHB	1 (6.7)	0 (0.0)	7 (10.6)	8 (8.3)
New onset LBBB	1 (6.7)	1 (6.7)	14 (21.2)	16 (16.7)
New onset AF	1 (6.7)	0 (0.0)	3 (4.5)	4 (4.2)
Poor wound healing	0 (0.0)	0 (0.0)	1 (1.5)	1 (1.0)
secondary thoracotomy for hemostasis	0 (0.0)	0 (0.0)	1 (1.5)	1 (1.0)
Coronary obstruction	0 (0.0)	0 (0.0)	2 (3.0)	2 (2.1)
Perivalvular leakage				
mild	5 (33.3)	2 (13.3)	9 (13.6)	16 (16.7)
moderate	1 (6.7)	2 (13.3)	3 (4.5)	6 (6.3)
Permanent pacemaker	1 (6.7)	0 (0.0)	7 (10.6)	8 (8.3)
Stroke	1 (6.7)	2 (13.3)	2 (3.0)	5 (5.2)
all-cause mortality	1 (6.7)	0 (0.0)	2 (3.0)	3 (3.1)

CHB: complete heart block; LBBB: left bundle branch block; AF: atrial fibrillation

## Discussion

The main findings of this study are the following: (i) patients who underwent TAVR achieved significant improvements in BNP, NYHA class, LVEDD, LVEF, and PASP across the three HF classes, with a more rapid and pronounced improvement in the HFrEF and HFmrEF groups; (ii) patients with HFrEF undergoing TAVR demonstrated a rapid improvement in left ventricular (LV) function and have a similar short-term clinical outcomes as compared to those with HFmrEF; (iii) the risk of periprocedural adverse events in patients with severely impaired LVEF appears to be comparable to patients with normal or mildly reduced LVEF.

In previous studies [15, 18, 19], AS patients were divided into two groups according to preoperative LVEF (TAVR) and their overall clinical outcomes were followed up. But our study divided patients into three groups (HFrEF, HFmrEF, HFpEF) according to latest HF guidelines. In a single center study [19], they investigated clinical outcomes of high-risk patients with severe AS undergoing transcatheter aortic valve implantation(TAVI) stratified by LVEF. It showed patients with LVEF ≤ 30% experienced a rapid improvement in LVEF associated with improved NYHA functional class at 30 days after TAVI. Webb et al. [20] study showed there was a significant improvement in LVEF after valve insertion. Another study [18] demonstrated the outcome of patients with low-EF severe AS following TAVR is as good as that of patients with preserved-EF. The results of our study are in keeping with those in the published literature. In our study, various echocardiographic parameters were analyzed comprehensively, which allowed us to investigate possible improvements in cardiac structure, in additional to LVEF changes. Meanwhile, echocardiographic data available 1 week and 1 month post-TAVR allowed for time-dependent changes to be assessed across different classes of HF. Patients who underwent TAVR achieved good recovery across the three HF classes, with a more rapid and pronounced improvement in the HFrEF and HFmrEF groups. According to our study, the improvement of LVEF and LVEDD in patients with HFrEF and HFmrEF improved more significantly, most probably due to a ceiling effect in HFpEF patients, limiting LVEF and LVEDD improvement beyond a certain point. In addition, TAVR can significantly reduce PASP, AVPG, V_max_ in AS patients with HF. In our study, we also found that compared to 1 week after TAVR, more patients in HFrEF group recovered their LVEF to ≥ 50% at 1 month post-TAVR. And TAVR can continuously improve LVEF of HFrEF patients. Valvular parameters improved in all classes patients suggesting an equal success rate. Several studies have demonstrated a symptomatic benefit of TAVR as reflected by changes in NYHA functional class [20–22]. Our study demonstrated there was a similarly strong improvement of at least one NYHA class in all three groups. There were no statistically between-group differences. Meanwhile, the symptoms of HF were improved and the quality of life was significantly improved. In addition to TAVR being beneficial for the treatment of symptoms of valvular heart failure, our study has demonstrated favorable effects on neurohormonal activation and reverse cardiac remodeling. In AS, myocardial wall stress induces the synthesis of BNP [23]. Previous studies have demonstrated a relation between the severity of AS, clinical symptoms and the release of this neurohormone [23]. SAVR decreases myocardial wall stress and thereby results in a significant reduction of BNP [24]. Our findings are in line with this observation and have demonstrated that TAVR also leads to a reduction in neurohormonal activation. Nevertheless, because the levels of BNP correlate with myocardial wall stress and the severity of aortic valve stenosis [23], the reduction of BNP in our study might be evidence of the beneficial effects of TAVR on myocardial function. The improvement of BNP and NYHA class were mirrored by a marked improvement in LVEDD, LVEF, and PASP across the three classes. Previous clinical studies [15, 25, 26] have reported non-significant differences in major outcomes(such as complication rates, short- or long-term survival, in-hospital mortality and cardiovascular mortality following TAVR) for patients with low versus high LVEF. In our study, the incidence of adverse events was low in the three groups, and we did not detect a significant increase in the incidence of adverse events in HFrEF group. Therefore, TAVR may be offered to patients with both severe AS and HFrEF.

Severe left ventricular dysfunction has long been established as a marker for poor outcomes after SAVR [27, 28]. Many of AS patients have reduced LVEF resulting in reluctance to offer aortic valve replacement [28]. The advent of TAVR has transformed the conventional management of such patients, with results revealing favorable hemodynamic changes with TAVR in comparison to SAVR [29]. Previous studies have been widely diverged and inconsistent regarding the effect of baseline EF on TAVR outcomes. In a study of 11,292 patients undergoing TAVR, systolic dysfunction (LVEF < 30%) was not significantly associated with higher rates of mortality [26]. This was further supported in the PARTNER trial, where results revealed there were no statistically significant differences in early and late mortality rates in AS patients, regardless of their pre-procedural LVEF [30]. Our findings showed similar early mortality and complication rates between across the spectrum of LVEF. However, the PARTNER 2 study demonstrated baseline LVEF was an independent predictor of 2-year cardiovascular mortality [31]. Possible explanations for these conflicting results may be the different EF values used as a cut-off for LV dysfunction or that patients may have concomitant pathologies causing HF, such as ischaemic cardiomyopathy, which will impact on their prognosis. The main clinical implication suggested by this finding involves the necessity of early intervention in patients with systolic dysfunction, with the use of higher LVEF cut-off values to improve their outcomes [32–34]. Although our data showed good recovery and it did not increased risk in patients with HFrEF after TAVR. This does not mean that we can ignore severe AS patients with mild systolic dysfunction. Previous studies showed low EF is a risk factor affecting the prognosis of patients with severe AS [14]. Chronic pressure overload as a result of severe AS causes increased wall stress and oxygen demand, followed by LV remodeling and compensatory hypertrophy, impairing diastolic function and promoting myocardial ischemia, ultimately resulting in fibrosis and irreversible damage to myocardial systolic function. Therefore, for patients with severe AS and mild LV systolic dysfunction particularly at early stages, timely intervention should be carried out to prevent irreversible severe LV functional damage leading to a serious decline in quality of life.

HFpEF is an entity with growing incidence and prevalence. So far, no significantly beneficial treatment has been established for HFpEF patients. Most therapeutic options of HFpEF focused on symptom control, which is in contrast to the situation of HFrEF [35]. HFpEF patients represents a more heterogeneous population, some of them have not developed to HFrEF yet and some already have severely symptomatic HF but would maintain a normal EF. So, we must distinguish HFpEF from HFrEF as well as other aetiologies that have different treatment strategies [36]. Of note, if the patient is in a mild stage before the development of HFrEF, early intervention is the key. In addition, factors functioning in the progression from this stage to HFrEF may be our therapeutic target [37]. Diagnosis of HFpEF relies on the presentation of objective evidence of cardiac dysfunction, physical examination, imaging data, blood testing results or echocardiography data. When these tests are inconclusive, invasive exercise testing is necessary. Treatment of HFpEF aims at controlling of cardiac congestion symptoms, management of comorbidities. Future research should focus on refining HFpEF into different phenotypes to achieve personalized treatment.

Our study has several limitations. Firstly, this is a single-center, retrospective study with selection bias, which further limits our analysis. Secondly, the limitations of the study are related to the small sample size, which may limit statistical power. Thirdly, this study has a short-follow up period and some loss of follow-up, which limits the ability to detect delayed complications and out of hospital mortality outcomes post-TAVR.

## Conclusions

Patients who underwent TAVR achieved significant improvements in BNP, NYHA class, LVEDD, LVEF, and PASP across the three HF classes, with a more rapid and pronounced improvement in the HFrEF and HFmrEF groups. Complication rates were low in the different HF groups. There is no significant increase in the risk of periprocedural complications in the HFrEF and HFmrEF groups.

## Data Availability

The datasets generated and analyzed during the present study are not publicly available because of the restrictions by the Affiliated Yantai Yuhuangding Hospital of Qingdao University, but are available from the corresponding author on reasonable request.

## Abbreviations

TAVR: Transcatheter aortic valve replacement
AS: Aortic stenosis
HF: Heart failure
HFrEF: Heart failure with reduced ejection fraction
HFmrEF: Heart failure with mildly reduced ejection fraction
HFpEF: Heart failure with preserved ejection fraction
BNP: B-type natriuretic peptide
NT-proBNP: N-terminal pro-B-type natriuretic peptide
SAVR: Surgical aortic valve replacement
LVEF: Left ventricular ejection fraction
CKD: Chronic kidney disease
CHD: Coronary heart disease
PCI: Percutaneous coronary intervention
CABG: Coronary artery bypass grafting
MI: Myocardial infarction
TIA: Transient ischemic attack
AF: Atrial fibrillation
CHF: Chronic heart failure
hsTnI: High-sensitivity Troponin I
LAAD: Left atrium anteroposterior diameter
LVEDD: Left ventricular end-diastolic dimension
RVAD: Right ventricular anteroposterior diameter
LVEDV: Left ventricular end-diastolic volume
AVPG: Aortic valve peak gradient
AVMG: Aortic valve mean gradient
Vmax: Peak aortic velocity
TR: Tricuspid regurgitation
PASP: Pulmonary artery systolic pressure
AR: Aortic regurgitation
BMI: Body mass index
CT: Computed tomography
LV: Left ventricular
TAVI: Transcatheter aortic valve implantation
